# Pharmacogenetics of colorectal cancer in a third-level hospital in Valencia

**DOI:** 10.1515/almed-2024-0146

**Published:** 2024-11-07

**Authors:** Ana Comes-Raga, Luis Sendra Gisbert, Goitzane Marcaida-Benito, Salvador F. Aliño Pellicer, María José Herrero Cervera

**Affiliations:** Department of Clinical Biochemistry, 16803 Consorcio Hospital General Universitario de Valencia, Valencia, Spain; Gene Therapy and Pharmacogenomics Research Group, Department of Pharmacology, Universitat de València and IIS La Fe, Valencia, Spain; Department of Pharmacology, University of Valencia and Pharmacogenetics Platform, IIS La Fe, Valencia, Spain

**Keywords:** pharmacogenetics, precision medicine, colorectal cancer, polymorphism, variant, genotyping

## Abstract

**Objectives:**

Genetic variants with associated pharmacokinetic and pharmacodynamic effects have an impact on the development of adverse drug reactions and survival of patients with colorectal cancer.

**Methods:**

A selection of genetic variants was performed according to the established chemotherapy and the pharmacogenetic databases. Genotyping was performed using MassArray technology (Agena Bioscience). Variant-toxicity and survival-genotype correlations were assessed using logistic regression (SPSS v.28.0.1.1).

**Results:**

Genotyping of 25 SNPs was performed in 96 patients. In relation to the *DPYD* gene, 3.5 % had the rs75017182 mutation; 4.7 % the rs1801158 mutation and 7.1 % the rs1801160 mutation. Genotypic frequencies in the *UGT1A1* gene were 39.4 % (*1/*1); 37.9 % (*1/*28); 19.7 % (*28/*28); and 3 % (*1/*36). The genotypes CT of the rs1801160 variant, AT of the rs67376798 variant (*DPYD*) and *1/*36 (*UGT1A1*) were associated with low survival (p-value: 0.006, <0.001, and 0.052, respectively). The most frequent adverse reactions were gastrointestinal disorders, followed by neurotoxicity. The CC genotype (rs1801160, *DPYD*) was associated with a lower risk for developing severe gastrointestinal events, whereas CC (rs1801158, *DPYD*) was associated with a lower risk of developing severe general hematologic toxicity.

**Conclusions:**

The population frequencies obtained in our study for rs1801160 and rs75017182 (*DPYD)*; and for *1/*28, *28/*, and *1/*36 (*UGT1A1)* were inconsistent with the frequencies reported for the Spanish population in the literature. The genotypes CT of rs1801160, AT of rs67376798 (*DPYD)*, and 1/*36 (*UGT1A1*) were associated with lower survival rates.

## Introduction

Pharmacogenetics is the study of the influence of individual genetic components on drug response and clinical outcomes. The effectiveness and toxicity of a particular drug are influenced by genetic variation among individuals [1].

Chemotherapeutic agents generally have a narrow therapeutic margin, and their effectiveness and safety are determined by individual variations in their metabolism. The approach to colorectal cancer (CRC) treatment is based on a range of chemotherapy options involving multiple drug combinations, with fluoropyrimidines being the cornerstone for treatment. Fluoropyrimidines are usually administered in combination with platinum compounds or irinotecan [2].

Fluoropyrimidines may cause severe adverse drug reactions, including mucositis, diarrhea and myelosuppression and, in 1 % of patients, they may cause death. This toxicity is frequently caused by the reduced activity of dihydropyrimidine dehydrogenase (DPD), the metabolic enzyme responsible for the breakdown of fluoropyrimidines that is encoded by the *DPYD* gene. Some DPYD mutations may reduce DPD activity, leading to previously described adverse events [3].

Platinum compounds (oxaliplatinum and cisplatinum) are used in combination with fluoropyrimidines for the treatment of CRC, and their use is not exempt from toxicity [4].

SN-38 is the active metabolite of irinotecan and a topoisomerase-I inhibitor, affecting DNA replication and transcription. The *UGT1A1* gene mediates the detoxification and conjugation of SN-38. Thus, mutations in this gene may cause toxicities including neutropenia, myelosuppression and diarrhea in patients receiving this treatment [5].

Platforms such as PharmGKB, the Clinical Pharmacogenetics Implementation Consortium (CPIC) and the Dutch Pharmacogenetics Working Group regularly conduct literature reviews and publish guidelines on the applications of pharmacogenetics in clinical practice. These efforts have contributed to a better understanding of pharmacokinetics and the genes associated with pharmacodynamics [6, 7]. Moreover, regulatory agencies such as the Food and Drug Administration (FDA) and the European Medicines Agency (EMA), provide pharmacogenetic information in the data sheets of the agents used for the treatment of CRC [8, 9].

In Spain, the Society of Pharmacogenetics and Pharmacogenomics (SEFF) and the Spanish Society of Medical Oncology (SEOM) have published clinical guidelines for the genotyping of six mutations of the *DPYD* gene in candidates to fluoropyrimidine-based chemotherapy [10].

In this study, genotyping was performed of genetic variants of the pharmacogenes involved in the metabolism of anticancer treatments in CRC. The study sample was composed of patients diagnosed with CRC in our Center. The objective of the study was to determine the frequency of these variants in our population and identify genetic biomarkers associated with the risk for developing adverse events and/or with reduced drug efficacy.

## Materials and methods

A retrospective observational study was carried out in which genetic variants involved in the metabolism of antineoplastic drugs for CRC were analyzed, in a sample of patients diagnosed with CRC in our Center (Consorcio Hospital General Universitario de Valencia, CHGUV). A literature search of publications and recommendations of the aforementioned organizations was performed to identify the variants involved in the metabolism of these agents. A total of 25 genetic variants were selected. The genes selected and their polymorphic variants are detailed in Table 1.

**Table 1:** Genes and mutations related to CRC pharmacogenetics analyzed in this research study.

Genes	Mutations	Level of evidence^a^
*DPYD*	rs3918290	1 A
	rs67376798	1 A
	rs55886062	1 A
	rs75017182	1 A
	rs115232898	1 A
	rs1801158	1 A
	rs1801160	1 A
	rs1801266	1 A
	rs1801268	1 A
	rs59086055	1 A
	rs777425216	NA
	rs78060119	1 A
	rs80081766	NA
*GSTP*	rs1695	3
*XPC*	rs2228001	3
*ERCC1*	rs3212986	3
	rs11615	3
*XRCC1*	rs25487	2 B
*UGT1A1*	*1/*1	1
	*28/*28	1
	*1/*28	1
	*1/*36	1
	rs4148323	1 B
*SEMAC*	rs7779029	3
*C8orf34*	rs1517114	3

^a^Based on the Pharmgkb database.

The sample was composed of patients >18 years diagnosed with CRC in our Center undergoing anticancer treatments, which completed a year of treatment and follow-up. Patients not undergoing anticancer treatments or refusing to take part in the study were excluded.

Study variables included demographics (age at diagnosis, sex, date of birth, and ethnicity); tumor-related data (date of diagnosis, tumor stage, presence/absence of metastases); treatment regimen received; adverse drug reactions (ADRs) and evaluation of response. All data was collected at the date of diagnosis, except for ADRs and the evaluation of response, which were collected one year after diagnosis.

ADRs were classified according to the Common Toxicity Criteria of Adverse Events (CTCAE v 4.03) [11]. ADRs were categorized as neurotoxicity, gastrointestinal (GI), hematologic, kidney, skin, and hepatic toxicity. ADR severity was also collected and categorized (mild: grade 1 and 2; severe≥3).

The oncologists followed the Response Evaluation Criteria In Solid Tumors (RECIST v1.1) [12].

The project was approved by the Ethics Committee of our Center. The samples collected and analyzed were composed of DNA extracted from whole blood drawn by venous puncture. SNPs were analyzed using the MassArray platform (Agena Bioscience).

The results obtained were analyzed using SPSS^®^ version 28.0.1.1. The correlation between SNPs and documented toxicities was assessed using logistic regression, statistical significance and odds ratios. The association between survival and the genotypes expressed in the population was examined using logistic regression for binary variables and Kaplan-Meier plotting. Survival was assessed one year after diagnosis of the neoplasm.

## Results

A total of 96 subjects were recruited, of whom 39 % were women and 56 % were men, with ages ranging from 28 to 83 years, and a median age of 67 years. All patients had Spanish nationality, except for one North American, three Latin Americans, one Russian, and one Bulgarian patient.

**Figure 1:**
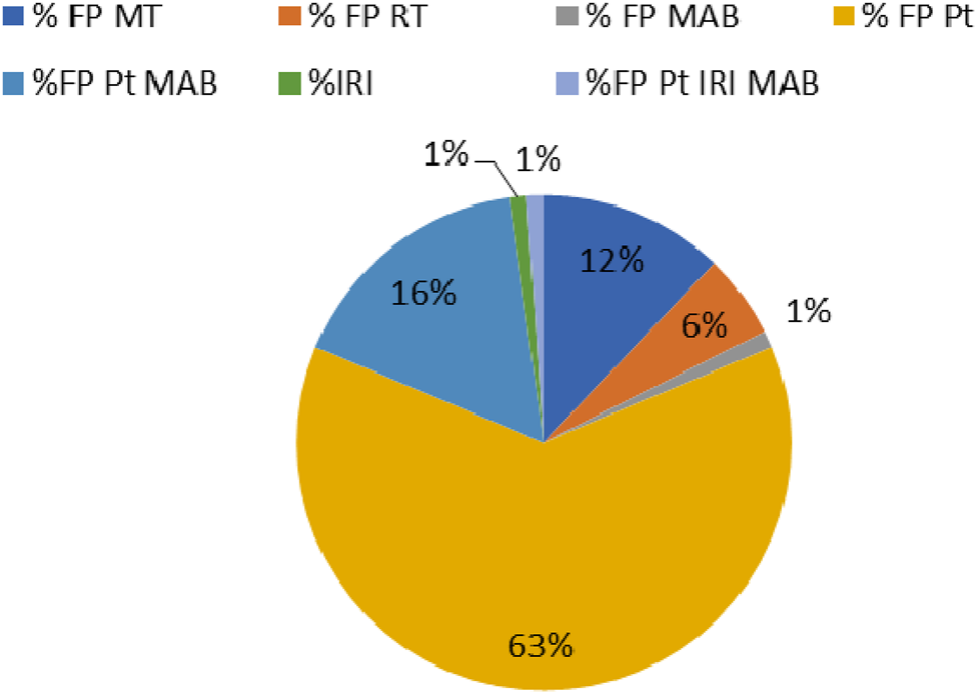
Graph of the therapeutic schemes used as first-line treatment in our study population. MAB (monoclonal antibodies): bevacizumab, and panitumumab; Pt (platinum compounds): cisplatin, oxaliplatin; FP (fluoropyrimidines): 5-fluorouracil, capecitabine; IRI (irinotecan); RT (radiotherapy); MT (monotherapy).

At diagnosis, 6 % of patients had stage-I CRC; 21 % had stage II CRC; 29 % had stage-III CRC, and 37 % had stage-IV CRC. As many as 20.4 % of patients had findings suggesting the presence of metastasis. The therapeutic regimens were based on fluoropyrimidines (5-Fluorouracil or Capecitabine) administered either, alone or in combination with radiotherapy, platinum compounds (Cisplatinum or Oxaliplatinum), and monoclonal antibodies (Bevacizumab or Panitumumab) and Irinotecan as add-on therapy. The different combinations are shown in Figure 1. In total, 34 % of patients received second-line treatment, whereas 6.6 % received three lines of treatment for their disease.

Screening for 25 SNPs was performed. The results are shown in Table 2, which includes all the genotypes frequency rates obtained, categorized by wild-type (WT), homozygous mutant (HomMut), and heterozygous mutant (HetMut).

**Table 2:** Genotyping results for our population. Results are expressed as % of carriers of the wild type genotype, heterozigous mutation (HetMut) and homozygous mutation (HomMut). Results for the *UGT1A1* gene are shown separately.

	Gen *DPYD*														
%	rs3918290(*2)	rs67376798-DPYD*14	Rs55886062(*13)	rs75017182G>C	rs56038477	rs115232898	rs1801158	rs1801160	rs1801266	rs1801268	rs59086055	rs777425216	rs78060119	rs80081766	rs9996584
Wild type	100	99	100	96.5	96.5	100	95.3	92.9	100	100	100	100	100	100	31.8
HetMut	0	1	0	3.5	3.5	0	4.7	7.1	0	0	0	0	0	0	51.8
HomMut	0	0	0	0	0	0	0	0	0	0	0	0	0	0	16.4

	**Gen *GSTP***	**Gen *XPC***	**Gen *ERCC1***	**Gen *XRCC1***	**Gen *ERCC1***	**Gen *SEMAC***	**Gen *C8orf34***	***UGT1A1***					
	rs1695	rs2228001	rs3212986	rs25487	rs11615	rs7779029	rs1517114	rs4148323	*1/*1	39.4					

Wild type	8.2	30.6	2.4	41.2	9.4	1.2	14.1	100	*28/*28	19.7					
HetMut	34.1	54.1	37.6	44.7	45.9	14.1	41.2	0	*1/*28	37.9					
HomMut	56.5	15.3	60.0	41.2	44.7	84.7	44.7	0	*1/*36	3.0					

The *DPYD* gene was identified in 3.5 % of HetMut patients for the rs75017182 mutation, in 4.7 % of patients with the rs1801158 mutation, and in 7.1 % of patients with the rs1801160 mutation.

With respect to the *UGT1A1* gene, 77.3 % of patients had the *1/*1 and *1/*28 genotype; 19.7 % had the *28/*28 genotype, and 3 % had the *1/*36 genotype. All patients had the WT genotype of the rs4148323 mutation.

In relation to the rs7779029 mutation in the *SEMAC* gene, 84.7 % of patients had the HomMut genotype; 14.1 % the HetMut genotype; and 1.2 % had the WT genotype. With respect to the rs1517114 mutation in the *C8orf34* gene, 14.1 % had the WT genotype, 41.2 % had the HetMut and 44.7 % had the HomMut genotype.

In relation to platinum compounds, screening was performed for the rs1695 mutations of the *GSTP* gene, with 8.2 % being WT, 34.1 % HetMut, and 56.5 % being HomMut; the rs2228001 mutation in the *XPC* gene (30.6 % WT, 54.1 % HetMut, and 15.3 % HomMut); the rs3212986 mutation in the *ERCC1* gene (2.4 % WT, 37.6 % HetMut, and 60 % HomMut); the rs25487 mutation in the *XRCC1* gene (41.2 % WT, 44.7 % HetMut, and 41.2 % HomMut); and the rs11615 mutation in the *ERCC1* gene (9.4 % WT, 45.9 % HetMut, and 44.7 % HomMut). All data is shown in Table 2.

**Figure 2:**
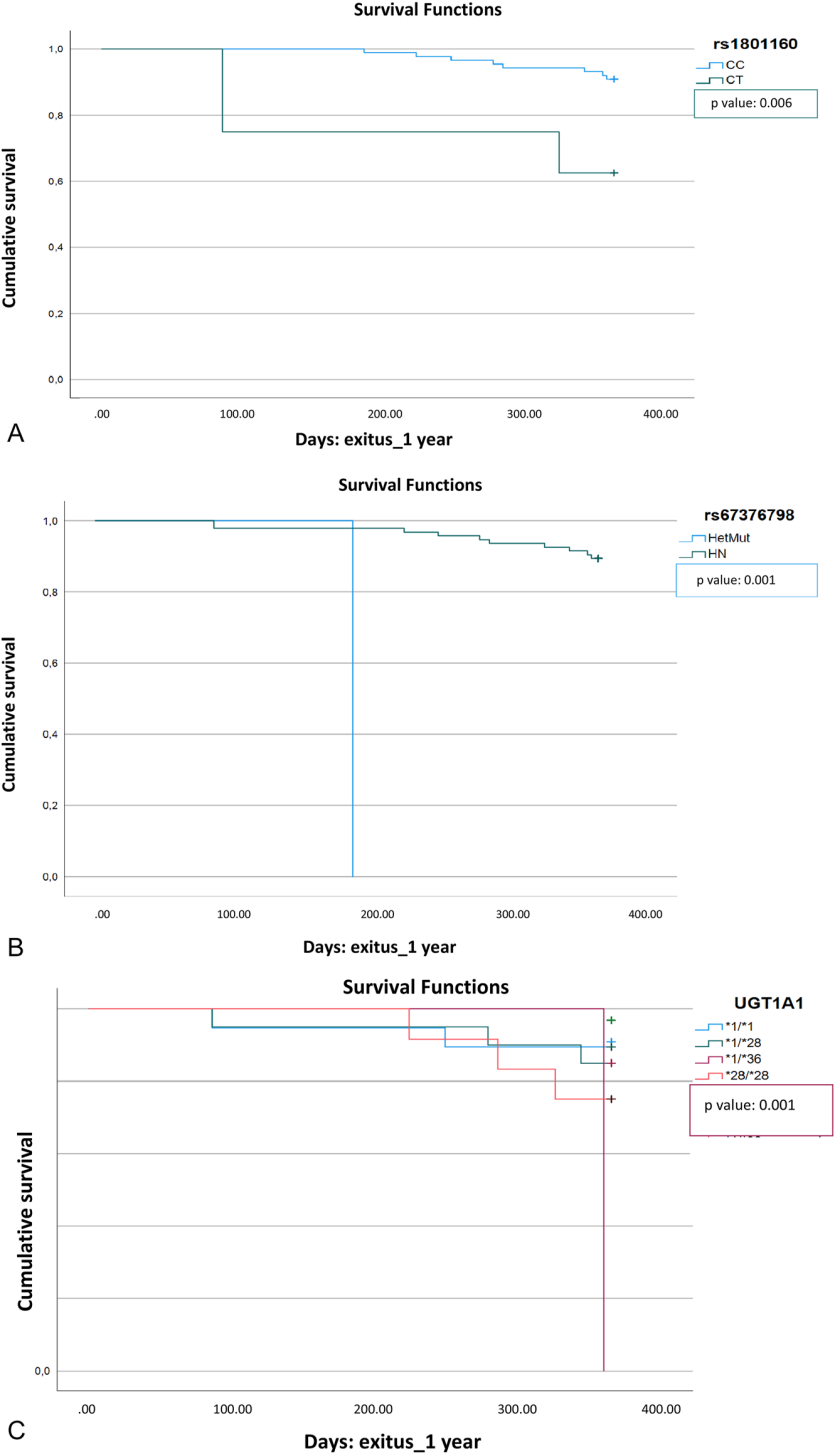
Graph displaying results from Kaplan-Meier survival plots. The graph on the left. (A) Represents the rs1801160 mutation (CC, CT genotypes); the graph in the middle (B) represents the rs67376798 mutation and the AT (heterozygous mutation) and wild type (TT) genotypes; the graph on the right (C) represents the *UGT1A* *1* gene and the mutations studied.

Regarding survival results for the SNPs studied, a statistically significant relationship was observed between genotypes CT of the rs1801160 mutation in the *DPYD* gene, AT (heterozygous mutation) of the rs67376798 mutation in the *DPYD* gene, and *1/*36 mutation in the *UGT1A1* gene (0.006, <0.001, and 0.052 respectively) with less survival. Survival curves are shown in Figure 2.

The following results were obtained from the ADRs study:

– Neurotoxicity: 42 % of patients developed neurotoxicity, with ADRs having been categorized as mild in 40.6 % of cases and severe in 59.3 %.
– Gastrointestinal reactions were reported for 68 % of our patients, being mild in 49 % of cases and severe in 51 %.
– Hematologic reactions were reported for 32 % of patients, with most being severe (75 %).

The association between the type of anticancer therapy and the ADRs developed by the patient is displayed in Table 3. All patients developed more than a type of ADR during the course of treatment. Gastrointestinal events were most frequent in fluoropyrimidines + radiotherapy; fluoropyrimidines + platinum compounds; and fluoropyrimidines + platinum compound + irinotecan + associated monoclonal antibodies. Gastrointestinal toxicities were as frequent as hematologic toxicities in fluoropyrimidines + associated monoclonal antibodies. Finally, patients developed the same rate of gastrointestinal and neurological reactions when ironotecan was administered.

**Table 3:** ADRs frequency rates by therapeutic scheme administered.

Therapeutic scheme	Hematologic toxicity, %		Gastrointestinal toxicity, %		Neurologic toxicity, %	
	Mild	Severe	Mild	Severe	Mild	Severe
Fluoropyrimidines (alone)	7.7	NA	38.5	7.7	46.2	NA
Fluoropyrimidines+radiation therapy	14.3	NA	28.6	42.9	14.3	NA
Fluoropyrimidines+MAB	50.0	NA	NA	50.0	NA	NA
Fluoropyrimidines+Platin C.	48.8	2.4	58.5	22.0	19.5	7.3
Fluoropyrimidines+Platin C.+MAB	46.2	3.8	46.2	19.2	21.1	7.7
Fluoropyrimidines+MAB+Irinotecan	NA	NA	100.0	NA	NA	NA
Irinotecan	NA	NA	NA	50.0	NA	50.0

NA, not applicable.

A relationship was observed between the CC genotype of the rs1801160 mutation in the *DPYD* gene and a lower risk for developing severe GI events (p-value<0.001; odds ratio 0.064). Likewise, the CC genotype of the rs1801158 mutation in the *DPYD* gene was also associated with a lower risk for developing all-type hematologic reactions (p-value<0.087; odds ratio 0.133) and severe hematologic reactions (p-value<0.001; odds ratio 0.058). This variant was classified as protective against severe hematologic adverse reactions.

No correlation was observed between the genotypes studied and neurological, kidney, skin and hepatic toxicities.

## Discussion

This study revealed that 15 % of CRC patients are carriers of a mutation in the *DPYD* gene. This mutation is associated with the loss of function of the DPD enzyme, resulting in an increased toxicity of fluoropyrimidine-based regimes. This rate exceeds the one reported in previous studies for the Spanish population, reporting a rate of 10 % [13].

The most frequent mutation was rs1801160 (six patients), followed by rs75017182 (five patients) and rs1801158 (four patients).

According to the literature, the rs1801160 mutation has a frequency rate of 5.6 % for the CT genotype, and 1.9 % for the TT genotype in the Caucasian population. In our series, the CT genotype had a frequency rate of 7.1 % andno cases of homozygous mutation (TT) have been reported to date.

The rs75017182 mutation is in linkage disequilibrium with rs56038477, with the two being related to the HapB3 haplotype. According to the evidence available for the Spanish population, the rs75017182 mutation has a population frequency of 3.7 %. In our series, frequency was 3.5 %. Since these two mutations are not always in a perfect linkage disequilibrium, screening for both mutations were performed in the entire study population [14].

In total, 4.7 % of our patients were carriers of the CT genotype of the rs1801158 mutation. None of our patients had the TT genotype. According to the literature, rates are 9.3 % and 0.9 % for the CT and TT genotypes, respectively.

According to the results obtained, the most frequent genotypes in our population were the CT genotype of the rs1801160 mutation and the GC genotype of the rs1801158, with a lower frequency of rs1801158, both in heterozygous and homozygous carriers.

For the rs1695 (*GSTP*), rs2228001 (*XPC*), rs3212986 (*ERCC1*), rs25487 (*XRCC1*), and rs11615 (*ERCC1*) mutations involved in the metabolism and action of platinum-based treatments, no statistically significant differences were observed between the genotypes of our series and the ones reported in the literature for the Spanish population [15].

Regarding the genes associated with response to irinotecan and its associated ADRs, some inconsistencies were detected in the frequency of mutations in the *UGT1A1* gene in the Spanish population. The *1/*1 mutation in our series had a frequency of 39.4 %, which is consistent with the one reported for the European population (40 %). The *28/*28 mutation had a frequency of 19.7 %, which exceeds the one reported for the Spanish population, 9 %. The *1/*28 genotype had a frequency of 37.9 % in our series, whereas the frequency described for the Spanish population was 51 %. Whereas not cases of *1/*36 have been reported in the literature, a carrier of this genotype was identified in our series (3.0 %) [16].

The rs1801158 and rs1801160 mutations of the *DPYD* gene were associated with a lower frequency of severe hematologic and GI events, respectively. Similar results have been reported for the rs1801158 mutation [17]. However, there is evidence in the literature of the role of the rs1801160 mutation in the development of severe GI adverse events, which is inconsistent with our results [18].

There is no evidence in the literature of an association between lower survival rate with the CT genotype of rs1801160 (p-value: 0.006) and the AT genotype of the rs67376798 (p-value<0.001) mutations in the DPYD gene, or 1/*36 in the UGT1A1 gene (p-value: 0.052).

Under the auspices of the Spanish Ministry of Health, all public hospitals in Spain offer pharmacogenetic services involving a panel of genetic tests. To date, the genes and mutations included for screening in the treatment of CRC are DPYD*2A, DPYD*13, *DPYD* c.2846A>T, *DPYD* c.1236G>A/HapB3; and *UGT1A1* [19]. The aim of this study was to provide evidence supporting the association between genetic mutations and phenotypes. These results may help improve the quality of personalized treatments.

It is necessary to perform a population study of the genes involved in the main metabolic pathways of the agents administered in CRC to ensure their appropriate administration.

### Limitations of the study

The results of this study were obtained during the course of a doctoral researcher’s PhD project. For the purposes of this project, other variables were collected that may have influenced the results obtained and should be examined in future studies.

These preliminary results should be tested and confirmed in a larger cohort study using more powerful statistical analyses to validate their use in clinical practice.
